# Agreement Between Provider-Completed and Patient-Completed Preoperative Frailty Screening Using the Clinical Risk Analysis Index: Cross-Sectional Questionnaire Study

**DOI:** 10.2196/66440

**Published:** 2025-02-10

**Authors:** Mehraneh Khalighi, Amy C Thomas, Karl J Brown, Katherine C Ritchey

**Affiliations:** 1 Division of General Internal Medicine Department of Medicine University of Washington Seattle, WA United States; 2 Hospital and Specialty Medicine VA Puget Sound Health Care System Seattle, WA United States; 3 Division of Geriatrics and Gerontology Department of Medicine University of Washington Seattle, WA United States; 4 Geriatric Research, Education and Clinical Center VA Puget Sound Health Care System Seattle, WA United States

**Keywords:** Risk Analysis Index, preoperative screening, questionnaire, frailty, self-reported, veteran, hip, knee, arthroplasty, elective surgery, cross-sectional, quality improvement

## Abstract

**Background:**

Frailty is associated with postoperative morbidity and mortality. Preoperative screening and management of persons with frailty improves postoperative outcomes. The Clinical Risk Analysis Index (RAI-C) is a validated provider-based screening tool for assessing frailty in presurgical populations. Patient self-screening for frailty may provide an alternative to provider-based screening if resources are limited; however, the agreement between these 2 methods has not been previously explored.

**Objective:**

The objective of our study was to examine provider-completed versus patient-completed RAI-C assessments to identify areas of disagreement between the 2 methods and inform best practices for RAI-C screening implementation.

**Methods:**

Orthopedic physicians and physician assistants completed the RAI-C assessment on veterans aged 65 years and older undergoing elective total joint arthroplasty (eg, total hip or knee arthroplasty) and documented scores into the electronic health record during their preoperative clinic evaluation. Participants were then mailed the same RAI-C form after preoperative evaluation and returned responses to study coordinators. Agreement between provider-completed and patient-completed RAI-C assessments and differences within individual domains were compared.

**Results:**

A total of 49 participants aged 65 years and older presenting for total joint arthroplasty underwent RAI-C assessment between November 2022 and August 2023. In total, 41% (20/49) of participants completed and returned an independent postvisit RAI-C assessment before surgery and within 180 days of their initial evaluation. There was a moderate but statistically significant correlation between provider-completed and patient-completed RAI-C assessments (*r*=0.62; 95% CI 0.25-0.83; *P*=.003). Provider-completed and patient-completed RAI-C assessments resulted in the same frailty classification in 60% (12/20) of participants, but 40% (8/20) of participants were reclassified to a more frail category based on patient-completed assessment. Agreement was the lowest between provider-completed and patient-completed screening questions regarding memory and activities of daily living.

**Conclusions:**

RAI-C had moderate agreement when completed by providers versus the participants themselves, with more than a third of patient-completed screens resulting in a higher frailty classification. Future studies will need to explore the differences between and accuracy of RAI-C screening approaches to inform best practices for preoperative RAI-C assessment implementation.

## Introduction

Frailty is a multidimensional syndrome characterized by decreased physiological reserve reducing recovery from stressors including surgery and is associated with increased postoperative morbidity and mortality [1]. Frailty screening and multidisciplinary management of persons with frailty before elective surgery improve perioperative functional performance, decrease postoperative mortality, and may improve postoperative morbidity [2,3]. While numerous patient-completed frailty screening tools (eg, FRAIL Scale, Edmonton Frail Scale, and Vulnerable Elders Survey) have been used to predict surgical morbidity and mortality in different surgical populations, few have undergone as extensive validation in the presurgical population as the Clinical Risk Analysis Index (RAI-C) [4,5]. The RAI-C is a validated 14-item health and functioning questionnaire developed to distinguish between frail and robust persons in the preoperative setting. It calculates a score between 0 and 81 from information provided by a person or surrogate with scores ≥37 indicating frailty [5-7]. Higher RAI-C scores have been associated with postoperative mortality across surgical specialties suggesting its use as an easily administered preoperative risk-stratification tool [6-8]. RAI-C has been adopted by the Veterans Health Administration (VHA) as the preferred tool for presurgical frailty assessment with the goal to optimize the care of at-risk persons [9].

Validation studies suggest that persons can complete the RAI-C independently, which is advantageous if provider time is limited [6-8]. However, review of study methods indicates that providers modified participant responses as needed, suggesting that screening was not entirely patient-led [6,7]. It is uncertain how often providers changed participant responses, which domains were modified, and how modifications affected frailty classifications. Therefore, we sought to examine provider-completed versus patient-completed RAI-C assessments to identify areas of disagreement between the 2 methods and inform best practices for RAI-C screening implementation.

## Methods

### Screening Procedures

#### Overview

As part of a quality improvement initiative, we designed and implemented a cross-sectional pilot examination to screen participants aged 65 years and older referred to an outpatient, VHA orthopedic clinic for elective total joint arthroplasty (TJA; eg, total hip or knee arthroplasty) for frailty between November 2022 and August 2023. The primary aim was to examine the agreement between provider-completed and patient-completed RAI-C assessments to inform frailty screening practices at our institution.

Orthopedic physicians and physician assistants underwent training on the use of an electronic health record (EHR)–embedded web-based RAI-C questionnaire. During preoperative evaluations, providers screened participants for frailty using the EHR-embedded assessment and recorded the RAI-C scores. Robust (RAI-C <30), prefrail (RAI-C 30-36), and frail (RAI-C ≥37) classifications were based on cutoffs defined in a large recalibration and external validation study of patients undergoing major elective noncardiac surgery. In that study, the 180-day postoperative mortality rate for RAI-C ≥30 was 2.0%, surpassing the overall mean mortality rate of 1.8%, and 4.3% for RAI-C ≥37, which is greater than twice the mean mortality rate of the population [5]. All participants also underwent screening for dementia with the Mini-Cog, a validated cognitive screen combining 3-item word memory and clock drawing [10]. Scores range from 0 to 5, with scores <3 indicating significant risk for dementia [11]. After the visit, participants were mailed a paper version of the RAI-C with a letter explaining the purpose of the screening tool and instructions on how to send back completed forms to study coordinators.

Participants were excluded from the study if surgery was performed before the participant responses to the RAI-C were received for analysis to mitigate possible confounding effects of surgery on patient-completed RAI-C responses. In addition, we excluded participant responses that were received more than 180 days from the date of provider-completed RAI-C to avoid confounding effects of progressive loss of function and osteoarthritis-related pain on the patient-completed RAI-C results. We chose an exclusion cutoff of 180 days based on findings that in individuals awaiting TJA for more than 180 days, worsening patient-reported outcome measures (ie, joint-specific function and health-related quality of life) were associated with increased levels of clinical frailty [12].

#### Analysis of Intervention and Measures

Patient-completed RAI-C responses were compared with provider-completed EHR-RAI-C results and analyzed for discrepancies between their total RAI-C and individual domain scores. The study authors performed a detailed EHR review to verify accuracy of provider and participant responses pertaining to health conditions (ie, presence of renal failure, heart failure, weight loss, or cancer). The provider completing the RAI-C also performed Mini-Cog screening for dementia to identify persons who would benefit from geriatric consultation (eg, scores <3), but results of this screening did not inform the subjective participant responses to the RAI-C question on loss of memory. The accuracy of participant responses to subjective questions (ie, limitations in activities of daily living [ADLs], loss of appetite, or memory problems) was not verified.

The primary outcome measure was the degree of concordance between provider-completed and patient-completed total RAI-C scores. Secondary outcome measures were degree of concordance between the responses for individual domains and the effect of time elapsed between provider-completed and patient-completed responses on the degree of concordance between scores. The Pearson product-moment correlation coefficient (*r*) was used to determine the linear relationship between provider-completed and patient-completed total RAI-C and individual RAI-C domain scores and time elapsed in days from provider to participant completion of the RAI-C and the absolute difference in scores obtained, respectively. Quantile-quantile plots and histograms of both the provider-completed and patient-completed total RAI-C scores indicated that the distributions of both variables were approximately normal. All analyses were performed in R (version 4.3.1; R Foundation for Statistical Computing).

### Ethical Considerations

The Human Research Protection Program, Associate Chief of Staff for Research and Development, and Quality, Safety, and Values department reviewed this project in accordance with the Veterans Health Administration Program Guide 1200.21 and determined that it was a nonresearch, operations activity. Thus, approval by an institutional review board and consent to participate were not needed. Participant data were anonymized to ensure privacy and confidentiality. Participants were not offered compensation.

## Results

Forty-nine participants aged 65 years and older presenting for TJA underwent RAI-C screening between November 2022 and August 2023. In total, 61% (30/49) of participants returned a postvisit RAI-C assessment, but 9 participants underwent surgery before completion and were excluded from analysis. An additional participant who returned a postvisit RAI-C assessment more than 180 days from orthopedic clinic evaluation was excluded. Therefore, 41% (20/49) of participants who returned a completed postvisit RAI-C assessment before surgery within 180 days from their initial evaluation were included in our analysis and their characteristics are summarized in Table 1. The number of positive responses to RAI-C questions reported in  Table 2 show all responses. Identical result counts between provider and patient responses do not necessarily indicate agreement between their respective responses.

**Figure 1:**
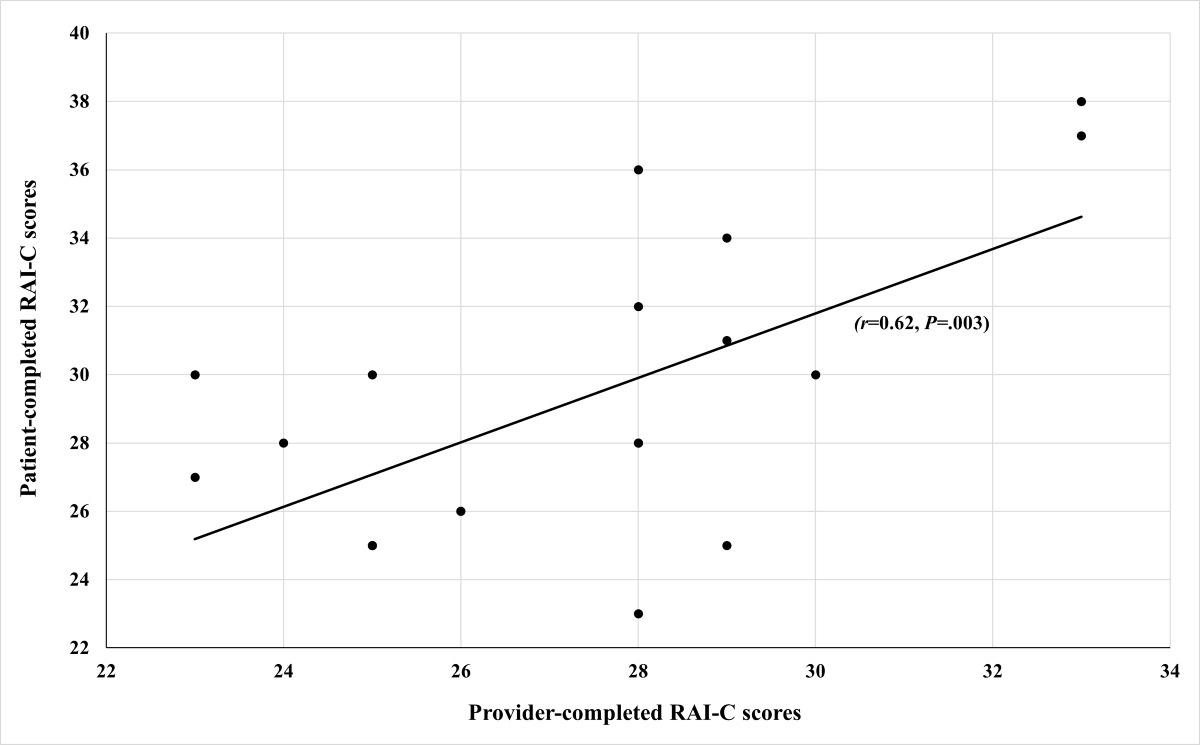
Correlation between provider-completed and patient-completed total RAI-C scores (N=20). RAI-C: Clinical Risk Analysis Index.

We used RAI-C score without cancer in our analysis since none of the participants met RAI-C definition of cancer (ie, unresectable cancer, metastatic cancer with poor prognosis, chemotherapy within 30 days, or radiotherapy within 90 days). There was statistically significant, moderate correlation between provider-completed and patient-completed RAI-C (N=20, *r*=0.62, 95% CI 0.25-0.83; *P*=.003; Figure 1).

**Table 1:** Participant characteristics (N=20).

Characteristics		Values	
**Gender, n (%)**			
	Men		19 (95)
Average age, years (range)			74 (66-83)
**Race, n (%)^a^**			
	White		17 (85)
	Black		2 (10)
**Preferred language, n (%)^b^**			
	English		18 (90)
Mini-Cog score ≥3, n (%)			19 (95)

^a^One participant declined to respond.

^b^Two participants declined to respond.

**Table 2:** Patients’ and providers’ responses.

Factors					Patient-completed			Provider-completed
**Medical conditions per RAI-C^a^ definition, n (%)**								
	Kidney disease			0 (0)			0 (0)	
	Heart failure			3 (15)			0 (0)	
	Shortness of breath			0 (0)			0 (0)	
	Cancer within 5 years			0 (0)			0 (0)	
**Nutrition, n (%)**								
	Loss of weight					3 (15)	1 (5)	
	Loss of appetite					1 (5)	0 (0)	
**Cognition, n (%)**								
		Loss of memory				4 (20)	3 (15)	
**Limitations in activities of daily living, n (%)**								
		Mobility				10 (50)	10 (50)	
		Eating				3 (15)	1 (5)	
		Toileting				2 (10)	0 (0)	
		Personal hygiene				2 (10)	0 (0)	
**Total RAI-C score, n (%)**								
			RAI-C <30 (Robust)			11 (55)	17 (85)	
			RAI-C 30-36 (Prefrail)			7 (35)	3 (15)	
			RAI-C ≥ 37 (Frail)			2 (10)	0 (0)	

^a^RAI-C: Clinical Risk Analysis Index.

Frailty classification was identical in 60% (12/20) of participants. The remaining 40% (8/20) of participants were reclassified to a higher level of frailty based on patient-completed RAI-C scores. In addition, 30% (6/20) of participants were reclassified from robust to prefrail and 10% (2/20) from prefrail to frail (Multimedia Appendix 1).

**Figure 2:**
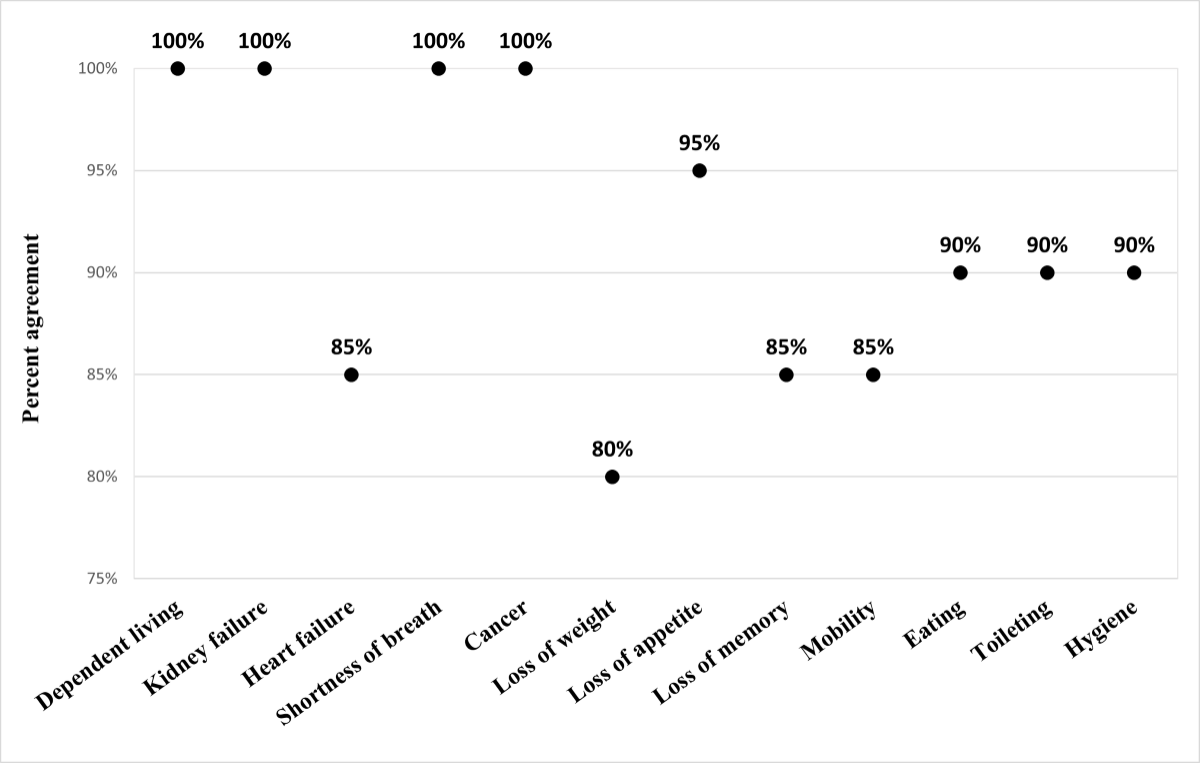
Percentage agreement between provider-completed and patient-completed responses to individual domains of the Clinical Risk Analysis Index (N=20).

Agreement between questions concerning chronic health conditions such as kidney disease and cancer was relatively high (Figure 2).

The domains with lowest agreements included heart failure, loss of weight, loss of memory, and the mobility subcategory of ADLs. Neither participant nor provider responses to weight loss (ie, loss of ≥10 lb in the past 3 months without trying) were accurate as they were not supported by EHR-documented weights. Although participant responses to weight loss compared with provider responses differed in 20% (4/20) of participants, this disagreement did not affect their respective frailty classification.

In responding to questions on loss of appetite, loss of memory, and limitations in ADLs, 45% (9/20) of participants assigned lower scores than providers, which reclassified 6 of these participants to a higher level of frailty. Therefore, participant responses to questions pertaining to loss of appetite, loss of memory, and ADLs accounted for 75% (6/8) of observed reclassifications to a higher level of frailty. The remaining 2 observed reclassifications to a higher level of frailty were based on participant responses indicating presence of heart failure, which was supported on review of EHR documentation of heart failure symptoms or consistent findings on transthoracic echocardiography.

On average, participants returned self-assessments within 41 days of the date the forms were mailed to them (median 28, range 21-68 days) with an average time between completion of provider and participant RAI-C forms of 65 (median 65, range 25-118) days. Time elapsed between assessments did not correlate with the differences observed between RAI-C scores (N=20, *r*=0.38; *P*=.10).

## Discussion

### Principal Findings

The RAI-C is preferred for preoperative frailty screening in VHA and has been validated in presurgical populations [5-7]. These prior validation studies have not fully explored the relationship between provider and patient-completed assessments as a method to increase screening efficiency. We showed that our population of older veterans with low concern for cognitive impairment presenting for elective orthopedic TJA could complete RAI-C assessments independently. However, the correlation between provider-completed and patient-completed RAI-C scores was only moderate and more than a third of participants were reclassified to higher levels of frailty based on self-assessment. While other studies comparing provider versus participant perceptions of frailty also observed moderate correlation between the 2 methods, their study populations and settings were different (emergency room vs preoperative setting), they used a different screening tool (Clinical Frail Scale vs RAI-C), and they found that providers assigned higher levels of frailty than participants [13,14]. Our study is one of the first to highlight areas of discrepancy between provider-completed and patient-completed RAI-C, suggesting challenges to the predictive validity of this tool and considerations for clinical implementation.

We found that disagreement between provider and participant responses and reclassifications were mainly based on participant-perceived decline in appetite, memory, and performance of ADLs, or heart failure. Notably, all participants accurately recognized their heart failure diagnosis, while providers missed the diagnosis in 3 cases. Disagreement between provider and participant responses to these domains (ie, heart failure, loss of appetite, memory loss, and limitations in ADLs) and provider underclassification of frailty has potentially significant clinical ramifications. Although optimal management of frailty is ill-defined, expert consensus suggests that persons with frailty should undergo comprehensive assessments to identify and address rehabilitative, nutritional, and psychosocial needs preoperatively [15]. Emerging data suggest that multimodal interventions can improve postsurgical outcomes for persons with frailty undergoing elective surgeries [3,16,17]. High-risk surgical candidates with frailty should have exploration of their health care priorities, postsurgical goals, and care preferences to avoid potentially deleterious postoperative outcomes [18]. Clarification of goals of care in the context of surgical risk and expected clinical outcomes, termed “surgical pause,” increases receipt of goal-concordant care and avoids unwanted surgery [19]. Thus, adequately and accurately identifying level of functional ability, cognition, and ultimately frailty of preoperative persons is important for unbiased care planning and resource allocation.

However, disuse or incorrect use of frailty screening tools can contribute to misclassification of frailty, potentially limiting access to interventions and significantly impacting quality of life and function. Elective TJA is rarely lifesaving but significantly impacts functional ability and preservation of independence [20]. Without consistent use of validated tools to screen for frailty, ageism and other implicit biases may contribute to overclassification of frailty by health care professionals and increase their reluctance to offer therapies simply based on biological age or “old” appearance [21]. Alternatively, concerns about surgical candidacy, unaddressed pain, and further loss of function may contribute to social desirability and response biases that encourage underclassification of frailty by participants who are reluctant to report functional or other limitations when responding to provider questions assessing for presurgical frailty [22,23]. Similar to responses to sensitive questions, where perceptions of anonymity and privacy increase the accuracy of self-reported answers, written responses to questions on performance of ADLs may be more accurate than verbal responses to providers, especially during the first encounter when participants have not yet built rapport with their providers [24,25].

In addition, the lived experiences of older adults and their perception of health may influence frailty classification and related health outcomes [26]. The person’s perception of decline in one domain (eg, performance of ADLs) may affect performance in other domains (eg, decline in appetite or memory) with a cumulative effect on level of frailty [26]. Therefore, the participants’ responses could be considered a more accurate reflection of subjective symptoms or functional ability, as they represent the individuals’ perceptions of their health.

When participants respond to the same questions without provider oversight, the effect of these biases may be minimized, and the accuracy of the screening tool might improve.

### Limitations

Our evaluation was limited to a small population of mainly English-speaking men with low concern for cognitive impairment within 1 VHA orthopedic surgery clinic which may not relate to other presurgical populations (eg, peripheral vascular surgery or general surgery) with different prevalences of frailty and cognitive impairment. In addition, worsening joint-specific function and health-related quality of life with longer wait times before TJA or surgical intervention between provider and patient-completed RAI-C can influence participants’ responses. Therefore, we attempted to mitigate possible confounding effects of prolonged wait times before surgery by excluding participant-completed RAI-C results that were completed more than 180 days from provider-completed surveys. We attempted to mitigate the effect of surgery on patient-completed RAI-C by excluding those participants who underwent surgery before completing the self-reported RAI-C. Nonetheless, our study was strengthened by the high participant response rate of more than 40%. In most cases of disagreement (ie, cognition and limitations in ADLs), participants’ responses resulted in a higher frailty classification, which could not be verified for accuracy. Furthermore, we could not assess for the role of selection bias on our findings. It is possible that self-reported responses to the RAI-C were predominantly completed and returned by participants who disagreed with provider-completed responses to the RAI-C. Finally, participant completion of the RAI-C relies on the ability to read and understand the questions. We were unable to assess the effects of health literacy or educational level on assessment disagreements.

### Conclusions

Frailty screening with the RAI-C can be done by providers or patients before elective orthopedic TJA. The level of disagreement observed between provider-completed and patient-completed assessments suggests that these methods are not interchangeable. Future studies exploring screening methods in larger, more diverse populations who are undergoing a variety of surgeries may clarify challenges to screening accuracy and validity of patient-completed screening approaches.

## Appendix

Multimedia Appendix 1 Frailty classification based on provider-completed versus patient-completed RAI-C scores (N=20). RAI-C: Clinical Risk Analysis Index.

## Abbreviations

ADLs: activities of daily living
EHR: electronic health record
RAI-C: Clinical Risk Analysis Index
TJA: total joint arthroplasty
VHA: Veterans Health Administration
