# Supplementary figures and images for: Agreement Between Provider-Completed and Patient-Completed Preoperative Frailty Screening Using the Clinical Risk Analysis Index: Cross-Sectional Questionnaire Study
Source: JMIR Perioper Med. 2025 Feb 10;8:e66440. doi: 10.2196/66440 (PMC11851030; doi:10.2196/66440)

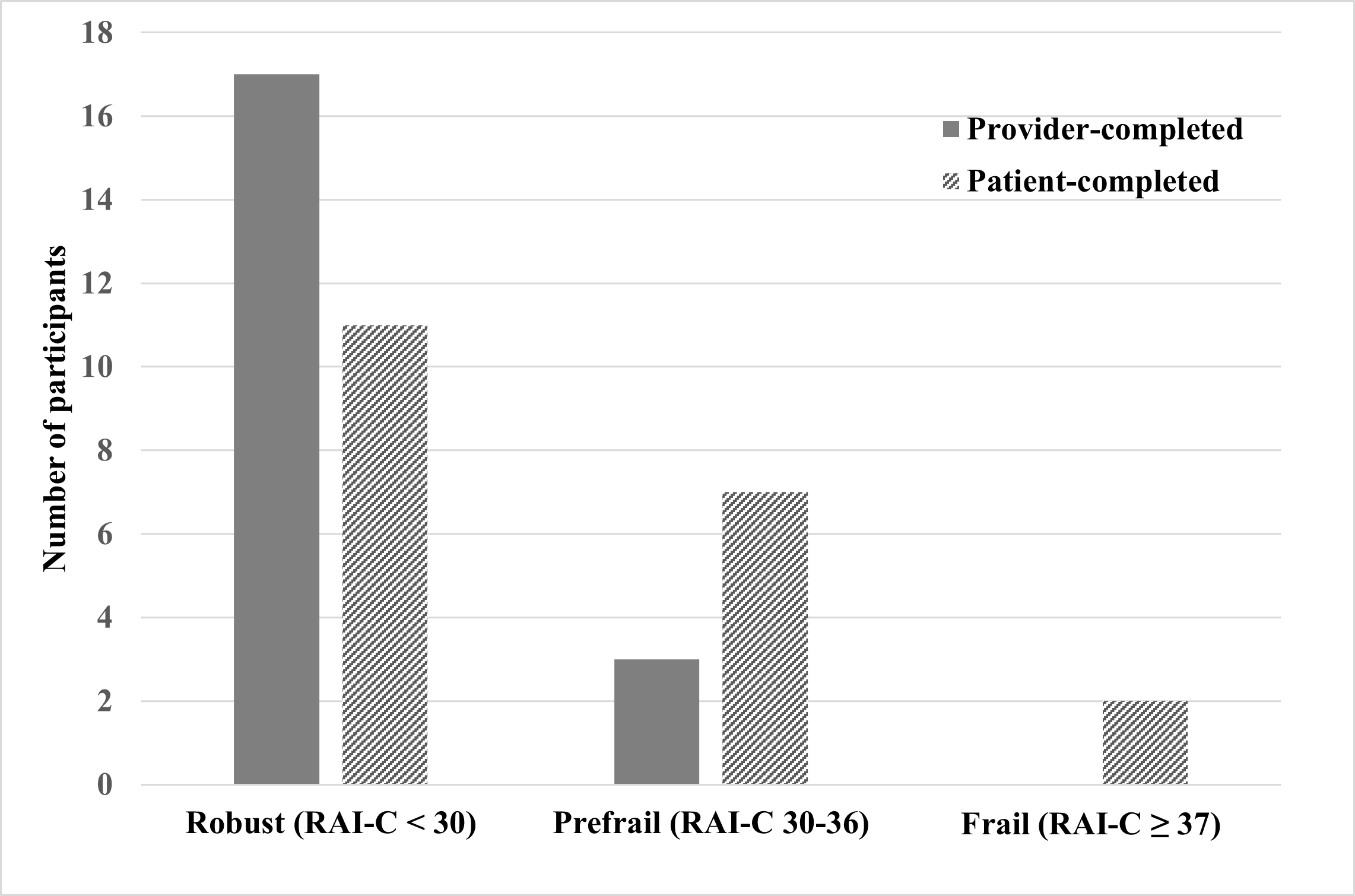

Supplement: Multimedia Appendix 1 [file periop_v8i1e66440_app1.png]
